# Fabrication of Tungsten Oxide Nanowalls through HFCVD for Improved Electrochemical Detection of Methylamine

**DOI:** 10.3390/mi15040441

**Published:** 2024-03-26

**Authors:** Mohammad Imran, Eun-Bi Kim, Tae-Geum Kim, Sadia Ameen, Mohammad Shaheer Akhtar, Dong-Heui Kwak

**Affiliations:** 1 Advanced Materials and Devices Laboratory, Department of Bio-Convergence Science, Jeonbuk National University, Jeongeup Campus, Jeongeup 56212, Republic of Korea; mohdimran@jbnu.ac.kr (M.I.); keb821@naver.com (E.-B.K.); 2 Environmental Engineering Laboratory, Department of Bioactive Material Sciences, Jeonbuk National University, Jeonju 54896, Republic of Korea; 3 Department of Bio-Convergence Science, Jeonbuk National University, Jeongeup Campus, Jeongeup 56212, Republic of Korea; tgkim@jbnu.ac.kr; 4 Graduate School of Integrated Energy-AI, Jeonbuk National University, Jeonju 54896, Republic of Korea; 5 New & Renewable Energy Material Development Center (NewREC), Jeonbuk National University, Jeonbuk 56332, Republic of Korea; 6 Department of JBNU-KIST Industry-Academia Convergence Research, Jeonbuk National University, Jeonju 54896, Republic of Korea

**Keywords:** WO_3_, HFCVD, nanowalls, methylamine, electrochemical sensor, cyclic voltammetry, linear sweep voltammetry

## Abstract

In this study, well-defined tungsten oxide (WO_3_) nanowall (NW) thin films were synthesized via a controlled hot filament chemical vapor deposition (HFCVD) technique and applied for electrochemical detection of methylamine toxic substances. Herein, for the thin-film growth by HFCVD, the temperature of tungsten (W) wire was held constant at ~1450 °C and gasification was performed by heating of W wire using varied substrate temperatures ranging from 350 °C to 450 °C. At an optimized growth temperature of 400 °C, well-defined and extremely dense WO_3_ nanowall-like structures were developed on a Si substrate. Structural, crystallographic, and compositional characterizations confirmed that the deposited WO_3_ thin films possessed monoclinic crystal structures of high crystal quality. For electrochemical sensing applications, WO_3_ NW thin film was used as an electrode, and cyclic voltammetry (CV) and linear sweep voltammetry (LSV) were measured with a wide concentration range of 20 μM~1 mM of methylamine. The fabricated electrochemical sensor achieved a sensitivity of ~183.65 μA mM^−1^ cm^−2^, a limit of detection (LOD) of ~20 μM and a quick response time of 10 s. Thus, the fabricated electrochemical sensor exhibited promising detection of methylamine with considerable stability and reproducibility.

## 1. Introduction

In today’s landscape, metal oxides have emerged as valuable assets across various industries, owing to their unique chemical, physical, and electronic characteristics. Metal oxides offer versatility and find applications in a wide range of sectors, including environmental remediation [1], medical technology [2], energy solutions [3], water purification [4], and personal care product development [5]. The use of metal oxide is anticipated to grow even further, underscoring their pivotal role in advancing innovation and addressing contemporary challenges [6].

Tungsten oxide (WO_3_) is a wide-band-gap metal oxide [7] which has good properties for numerous applications, e.g., information science, electronics (nano and micro), computer science, energy (renewable and non-renewable), transportation, safety engineering, military technologies, optoelectronic [8], electrochromic devices [9] and sensing [10]. WO_3_ is an n-type semiconductor which exhibits high stability, small diffusion length (150–500 nm) and good carrier mobility (~33.9 cm^2^ V^−1^ s^−1^) [11]. Tungsten oxide (WO_3_) has become increasingly popular due to its superior sensitivity, heightened responsiveness to sensitization, excellent stability, and suitability for lower-temperature operations [12]. In thin-film configuration, WO_3_ offers optimal electrical resistivity, selectivity, and repeatability, making it a promising candidate in sensor technology. Furthermore, it exhibits the ability to undergo changes in characteristics such as stoichiometry, composition, structure, thickness, and morphology, depending on the synthesis techniques and conditions employed [13]. WO_3_ predominantly in thin films shows commendable performances due to its cost-effective properties, simple fabrication, and easy deposition process.

Various deposition techniques (vacuum and non-vacuum techniques) have been used to deposit nanostructured WO_3_ thin films on substrate surfaces, including pulsed laser deposition (PLD) [14]; the electrophoresis deposition process (EDP) [15]; sputtering [16]; chemical spray pyrolysis [17]; solvothermal [18], hydrothermal [19,20], and sol–gel methods [21]; the chemical vapor deposition method [22]; and the physical vapor deposition method [23,24]. Hot filament chemical vapor deposition (HFCVD) is a cost-effective process compared to other deposition techniques. Its economic benefits arise from the use of a hot filament that decomposes preceding gases, which enables effective film development at lower temperatures [25]. It significantly minimizes the consumption of energy and instrument costs. For thin-film deposition, HFCVD is capable of depositing thin films with a high surface-to-volume ratio and intricate architectures. Accurate control enables the production of high-resolution patterns for device electrode fabrication [26]. HFCVD is beneficial for the fabrication of sensor electrodes because it can control the electrical resistivity, selectivity, and durability of thin films [27,28].

Methylamine is a hazardous organic chemical found in liquid and gas forms [29]. It is toxic, colorless, and flammable at room temperature with a typical pungent smell [30]. Methylamine is used as industrial raw material for the production of pesticides as an agriculture product, making rubbers for transportation industries, dyes for textile industries, and in pharmaceutical industries [31]. It is a hazardous chemical, and its accidental release or exposure can pose risks to public health and safety. Thus, it is crucial to monitor the presence of methylamine to ensure safety and prevent potential accidents or exposures [32]. In this regard, electrochemical detection methods can provide real-time detection and monitoring [33] of methylamine, allowing for prompt action to mitigate any risks. In particular, the chemical sensing methods enable the detection and quantification of methylamine in environmental samples [34], helping to identify potential pollution sources and assess the impact on the environment [35,36].

With these motivations, in this work, we have deposited WO_3_ nanostructured thin film through HFCVD, using tungsten (W) filament in a constant O_2_ pressure. The unique morphology displays WO_3_ thin-film nanowalls (WO_3_ NWs) with a grain size of 20–25 nm. To the best of our knowledge, this is the first report on single-step HFCVD-deposited WO_3_ thin film with distinct morphology and has been used as an electrode material for the detection of hazardous methylamine.

## 2. Materials and Methods

### 2.1. Materials

Methylamine (CH_3_NH_2_, Sigma Aldrich, ≥99.5%, St. Louis, MO, USA), silicon (Si, p-type, 10 mm × 10 mm, Siltron Inc., Seoul, Republic of Korea), tungsten (W) wire (thickness ~0.5 mm, The Nilaco Corporation, Tokyo, Japan), sodium dihydrogen phosphate (NaH_2_PO_4_, Sigma Aldrich, ≥99%, St. Louis, MO, USA), and disodium hydrogen phosphate (Na_2_HPO_4_, Sigma Aldrich, ≥99%, St. Louis, MO, USA) were used.

### 2.2. Synthesis of WO_3_ NW Thin Film

**Figure 1:**
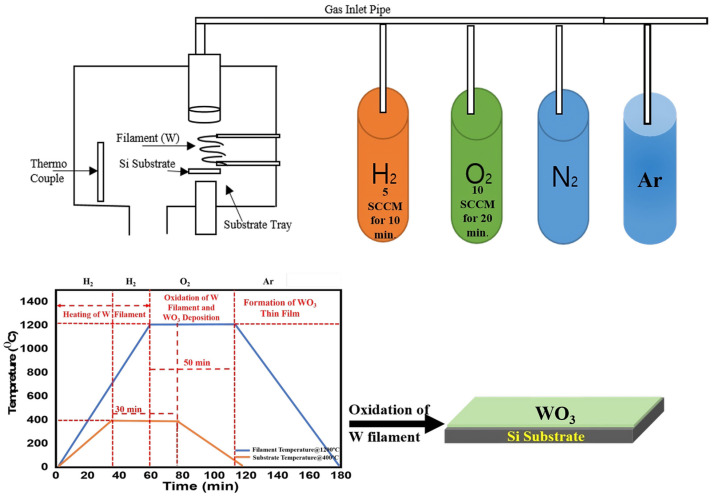
A schematic representation of the HFCVD process for the synthesis of WO_3_ NW thin films.

The WO_3_ NW thin film on the Si substrate was deposited through the HFCVD technique. As shown in Figure 1, the vacuum chamber is equipped with a thermocouple for measuring and monitoring the temperature, tungsten (W) wire filament was used, gas inlet pipes connected to mass flow controllers were used to regulate the flow of the gasses, and a high-current power supply, a rotary vacuum pump, and a cooling facility were attached to the vacuum chamber. For thin-film deposition, the silicon wafers (Si-P100) were cleaned with ultrasonic vibration using deionized (DI) water and acetone. Thereafter, Si substrates were placed on the substrate tray at the distance of ~10 mm from the W wire filament, and the chamber pressure was set to a constant 0.2 Torr. Subsequently, the W filament was heated at 1400 °C and the substrate temperature was raised from 300 to 450 °C for ~30 min under the supply of oxygen at a flow rate of 10 sccm. Herein, hydrogen gas was used as a precursor gas at 5.0 sccm. Finally, the W filament was oxidized at the above temperatures, resulting in the growth of WO_3_ nanostructured thin film on the Si substrate.

### 2.3. Characterization Techniques

Morphological analysis was carried out by a field-emission scanning electron microscope (FESEM, Hitachi S-4700, Tokyo, Japan). The elemental composition, mapping and line scan mapping analysis were determined by the SEM-coupled energy-dispersive X-rays spectroscopy (EDS). The absorption properties were obtained through a UV–visible spectrophotometer (JASCO, V-670, Tokyo, Japan). The structural investigations of WO_3_ nanostructured thin film were performed by X-ray diffraction (XRD, Rigaku, Tokyo, Japan) in the Bragg angle ranging between 20° and 60° to explain the crystal phases and lattice properties using CuKα radiation (λ = 1.5406 Å). Fourier-transform infrared (FTIR, Nicolet, IR300, Wisconsin, USA) was used to study bond vibrations in the range of 400–4000 cm^−1^. X-ray photoelectron spectroscopy (XPS, KRATOS AXIS-Nova, Manchester, UK) was conducted to study the surface compositional and element states.

### 2.4. Sensing Performance

To detect the presence of the hazardous methylamine, a three-electrode system of 10 mL electrochemical cells was employed for the measurement of cyclic voltammetry and linear sweep voltammetry using an electrometer (Keithley, 6517A, Aurora Rd, USA), Herein, HFCVD-grown WO_3_ NW thin film served as the working electrode, AgCl/Ag was employed as a reference electrode and gold wire was utilized as the counter electrode. A targeted analyte (methylamine) was prepared at a broad concentration range of 20 μM–1 mM in a 10 mL solution of 0.1 M phosphate buffer solution (PBS) of pH 7. The use of PBS in electrochemical sensing offers advantages such as stable pH, biocompatibility, consistent ionic strength, enhanced solubility, and compatibility. Its buffering capacity and versatility make PBS a reliable choice for maintaining optimal conditions during electrochemical measurements [37]. Cyclic voltammetry was performed to study the oxidation and reduction peaks and the linear sweep voltammetry technique was used to study the current and voltage responses. The active area of the fabricated electrode was 1 cm^2^ (WO_3_ NWs) and the sensitivity was calculated by dividing the slope of the calibrated current–concentration plot by the active area of the sensor, as expressed in Equation (1):

(1) $$\mathrm{Sensitivity}=\frac{\mathrm{Slope}\mathrm{of}\mathrm{calibrated}\mathrm{curve}}{\mathrm{Active}\mathrm{area}}$$

Herein, CV was performed within the range of −0.8~0.8 V, with a scan rate of 50 mV/s. The current responses were analyzed within a voltage range from 0 to 2.0 V, and the response time was determined to be 10 s.

## 3. Results

### 3.1. Morphological Properties of WO_3_ NW Thin Film

**Figure 2:**
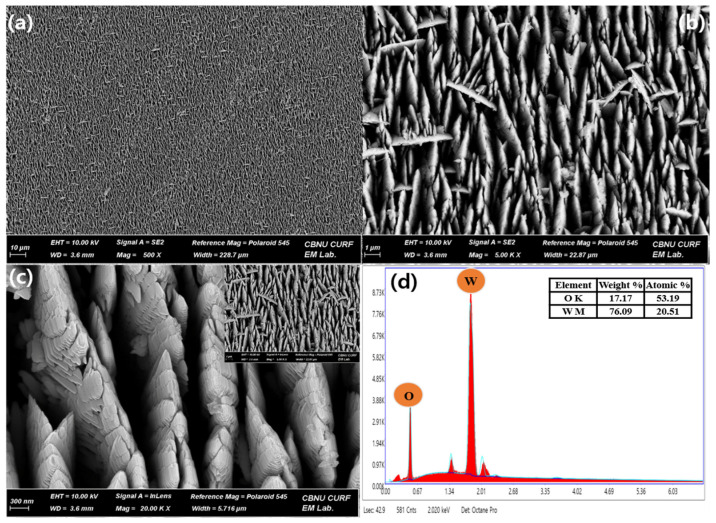
FESEM images (**a**–**c**) and EDX spectrum (**d**) of the HFCVD-grown WO_3_ NW thin film.

The morphology of HFCVD-grown WO_3_ nanostructured thin films deposited on the Si substrate was investigated by FESEM and the elemental characterization was performed by energy-dispersive X-ray (EDAX). The HFCVD-grown WO_3_ thin film, as shown in Figure 2a–c, depicts the formation of highly dense and uniformly distributed nanowall (NW) structures. At an optimized substrate temperature (Ts) of ~400 °C, the surface of the thin film appears notably uniform, with a grain size in the range of 20–25 nm. However, upon raising the substrate temperature, the grain size increase might be due to the increased reaction rate at the substrate surface [38]. This improved grain size might boost the overall surface area of the WO_3_ thin film, indicating the availability of more active sites for chemical interactions. In the context of chemical sensing, the large surface area of the thin film brings about enhanced sensitivity, a faster response time, a low limit of detection and increases the reproducibility of the targeted electrode [39,40]. Figure 2d shows an elemental analysis of the HFCVD-grown WO_3_ NW thin film, exhibiting the presence of two primary elements: W (tungsten) and O (oxygen). The EDX analysis quantifies the elemental composition, with tungsten accounting for ~24.51% and oxygen making up the remaining ~75.49%.

### 3.2. Optical Characterizations of WO_3_ NW Thin Film

**Figure 3:**
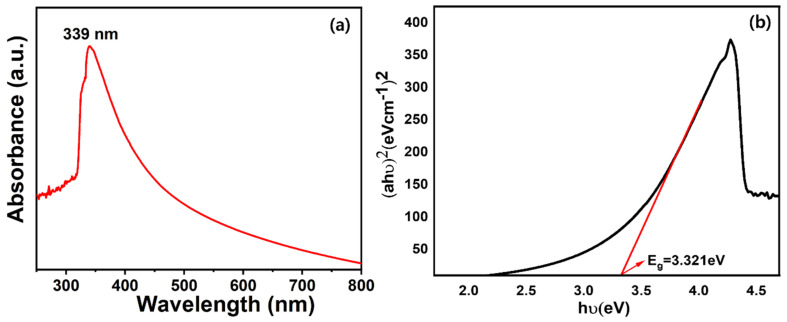
UV–vis spectroscopy (**a**) and its corresponding Tauc plot (**b**) of the HFCVD-grown WO_3_ NW thin film.

The optical properties of HFCVD-grown WO_3_ NW thin film are studied by UV–vis absorption in the range of 200~800 nm. The UV–vis spectrum, as shown in Figure 3a, exhibits a sharp absorption peak at ~339 nm, which confirms the deposition of the WO_3_ thin film [41,42]. The band gap value of WO_3_ NW thin film is calculated by the Tauc relation:

(2) $$\alpha =\frac{A{\left(h\upsilon -Eg\right)}^{2}}{h\upsilon }$$

where A, hυ and Eg are a constant of proportionality, photon energy and optical bandgap energy [43], respectively. In our work, the WO_3_ NW thin film has a band gap of ~3.321 eV, as shown in Figure 3b. The existence of an optical band gap energy of ~3.321 eV represents the minimum energy required to move an electron from the valence band (the highest energy band filled with electrons at absolute zero) to the conduction band (the lowest energy band with available electron states) within the thin film [44].

### 3.3. Crystalline and Structural Studies of WO_3_ NW Thin Film

**Figure 4:**
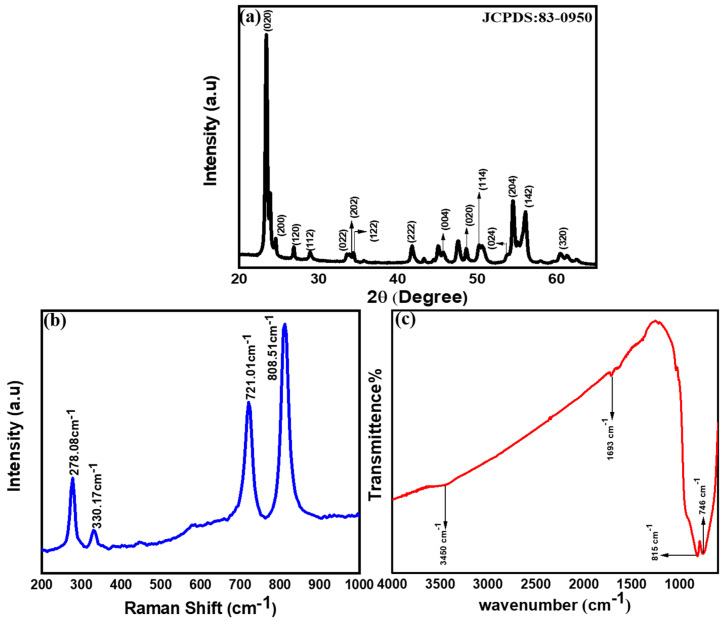
The XRD patterns (**a**), Raman spectrum (**b**) and FTIR spectrum (**c**) of HFCVD-grown WO_3_ NW thin films.

XRD is performed to study the crystal structures, phases, crystallite size, and purity of the synthesized WO_3_ NW thin film. Figure 4a exhibits the diffraction patterns at 23.40°, 24.54°, 26.84°, 28.91°, 33.59°, 33.89°, 34.46°, 41.78°, 45.83°, 48.61°, 50.27°, 53.78°, 54.45°, 56.09°, and 60.53° relating to miller planes (020), (200), (120), (112), (022), (202), (122), (222), (004), (020), (114), (024), (204), (142), and (320), respectively [45,46]. The obtained XRD diffraction peaks are well matched with JCPDS card no. 83-0950. The crystal size of WO_3_ NW thin film is calculated as ~83.3472 nm by the Scherrer formula [47], using the most intense peak at 23.40°.

(3) $$D=\left(\frac{k\lambda }{\beta cos\theta }\right)$$

where D is the crystallite size of the particle in nm, θ is the diffraction angle, β is the full width at half maximum observed in radians (FWHM), k is the Scherrer constant (k = 0.94) and λ is the X-ray wavelength (λ = 1.54178 Å) [24,25]. Herein, the WO_3_ NW thin film prepared at Ts of ~400 °C shows phase purity at the strongest diffraction peak of 020 lattice planes, indicating the preferential growth orientation [48].

Figure 4b displays the results of Raman spectroscopy, performed in the range of 200–1000 cm^−1^, to measure the structural and molecular properties of the WO_3_ NW thin film. Raman spectra clearly manifest the signature peaks associated with stoichiometric WO_3_ with a monoclinic phase [49]. The W-O-W stretching and bending vibrations are located between 700 and 800 cm^−1^ [48] and O-W-O stretching and bending vibrations are noticed between 250 and 400 cm^−1^ [49]. The intense band at ~278.08 cm^−1^ and the weak band at ~330.17 cm^−1^ are attributed to the bending vibration of δ(O-W-O) [50]. The Raman peak observed at ~801 cm^−1^ is the typical polycrystalline WO_3_ in the monoclinic or triclinic crystalline phase [48,49].

Chemical configurations of HFCVD-grown WO_3_ NW thin films at Ts of ~400 °C is shown in Figure 4c. The spectrum exhibits transmittance in the range of 400–4000 cm^−1^. The FTIR spectra exhibit a broad peak at ~746 cm^−1^ and 815 cm^−1^ [51], which are attributed to the stretching vibration of υ(O-W-O) [51]. The υ(O-W-O) stretching vibration mode is the monoclinic crystal phase, confirming that the WO_3_ NW thin film is grown well on the Si substrate [52]. The transmittance peaks located at ~746 cm^−1^ refer to the O-W-O bending mode of the vibration and transmittance peak located at ~846 cm^−1^ is due to the W-O stretching mode of hexagonal WO, confirming the hexagonal structure [53].

### 3.4. XPS Studies of WO_3_ NW Thin Films

**Figure 5:**
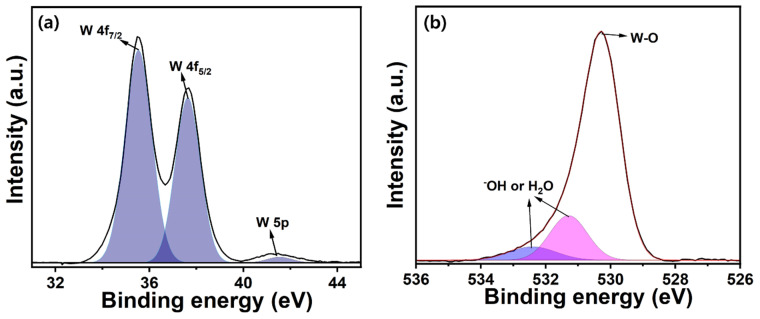
W 4f (**a**) and O 1s (**b**) XPS plots of HFCVD-grown WO_3_ NW thin films.

The XPS properties provide an in-depth understanding of the oxidation states of W and O in WO_3_ thin films using high-resolution spectra of W 4f and O 1s binding energies. Figure 5a showcases the fitted W4f XPS spectrum, revealing a distinct doublet pattern, with binding energies of approximately ~35.5 and ~37.7 eV [54]. These values correspond to the W 4f_5/2_ and W 4f_7/2_ electronic states, respectively. Peaks at 4f_5/2_ correspond to the binding energy of ~35.58 eV, aligning precisely with the characteristic energy level of the W^6+^ oxidation state within WO_3_ thin films and indicating the predominant oxidation state of tungsten [55] in the HFCVD-grown WO_3_ NW thin film. This peak serves as compelling evidence affirming that the WO_3_ thin film predominantly comprise hexavalent tungsten [56]. The second distinct XPS peak emerges at approximately ~37.9 eV, corresponding to the 4f_7/2_ state of tungsten ions in the HFCVD-grown WO_3_ NW thin film. This peak reaffirms the dominance of the W^6+^ oxidation state, which is crucial for understanding the electronic structure and chemical environment of the material [29,57]. Mostly, WO_3_ thin films are characterized by the presence of two distinct binding energies within the range of ~36–38 eV. These binding energies are indicative of the prevalence of W^6+^ ions within stoichiometric WO_3_ [25]. In our work, we observe a similar doublet pattern within the range of 36–38 eV, affirming the stoichiometric nature of the thin film [58,59]. The O 1s XPS plot, as depicted in Figure 5b, reveals two distinct binding energies: one with a higher intensity at ~530.4 eV and another with a lower intensity at ~531.4 and ~532.3 eV. Herein, the higher-intensity peak at ~530.2 eV typically arises from the O component of oxide, providing further evidence for the formation of W-O bonds in HFCVD-grown WO_3_ NW thin films [60]. Notably, the lower-energy peaks at ~531.4 and ~532.3 eV are associated with -OH or H_2_O species on the thin film’s surface, due to contamination that might occur with atmospheric moisture or crystal water [61].

### 3.5. Sensing Parameters of WO_3_ NW Thin-Film-Based Electrodes

**Figure 6:**
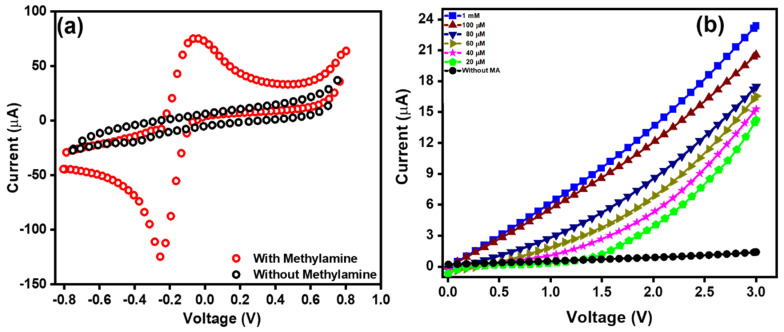
(**a**) Cyclic voltammetry plot with methylamine (1 mM) in 0.1 M PBS of a HFCVD-grown nanostructured WO_3_ thin film and (**b**) I–V curves of the HFCVD-grown nanostructured WO_3_ thin-film-based chemical.

A HFCVD-grown WO_3_ NW thin film-modified electrode is employed for the detection of methylamine. The electrochemical sensing studies were performed in 0.1 M phosphate buffer solution (PBS, pH = 7). Cyclic voltammetry (CV) measurements were used to analyze the electrochemical behavior at a scan rate of 50 mVs^−1^. The CV graphs for the detection of methylamine are shown in Figure 6a, exhibiting a promising electrochemical reversibility and efficiency with a redox response (oxidation and reduction) of the WO_3_ NW thin-film electrode of 0.2303 V redox potential [62]. These peaks usually occur due to the transfer of electrons displaying, the oxidation peak as a result of electron-losing behavior and the reduction peak occurs due to the electron-gaining behavior. In our work, the detection of methylamine by the HFCVD-grown WO_3_ NW thin-film electrode exhibits efficient electro chemical sensing behavior, which might be due to the high conductivity [33] and large surface area [63] of WO_3_ NW thin films. From linear sweep voltammetry, as shown in Figure 6b, a low current value of ~1.3 μA is observed in pristine PBS. However, it has been observed that upon the addition of the lowest amount of methylamine (20 μM), there is a significant change in current due to the high sensing properties of the HFCVD-grown WO_3_ NW thin film. Electrolytes with different concentrations of methylamine (20 μM–1 mM) display a lower current response of ~14.15 μA and the highest current value of ~23.23 μA. This gradual increase in the current indicates the rapid sensing response of HFCVD-grown WO_3_ NW thin-film electrodes in the detection of methylamine, which might result from the better electrocatalytic or electrochemical behavior and the fast electron exchange of HFCVD-grown WO_3_ NW thin films [64]. The increase in current upon the further addition of the targeted chemical in an electrolyte usually results in an increase in the ionic strength of the electrolyte. Herein, when increasing the methylamine concentration in the 0.1 M PBS, a large number of ions are generated; due to this response, more electrons are exchanged and this increases the opportunity for electron transfer at the electrode surface. As such, there is an increase in the rate of reaction, which enhances the sensitivity of the electrode. This result suggests that HFCVD-grown WO_3_ NWs show effective sensing capabilities in the detection of methylamine [65].

**Figure 7:**
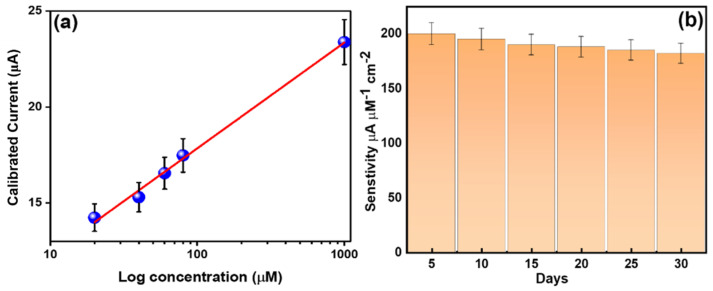
(**a**) Calibrated current versus the concentration of methylamine of HFCVD-grown WO_3_ NW thin-film-based chemical sensors. (**b**) Stability test through I–V measurements in the presence of 20 μM methylamine in 0.1 M PBS.

To examine the sensitivity of HFCVD-grown WO_3_ NW thin-film-based electrodes, Figure 7a shows the current vs. concentration calibration curve [33]. Herein, the current increases linearly with an increase in analyte concentration. The HFCVD-deposited WO_3_ NW thin-film-based electrode shows a reproducible, reliable and considerable promising sensitivity of ~183.65 μA mM^−1^ cm^−2^ with a linearity of 20 μM^−1^ mM, a detection limit of ~20 μM and a correlation coefficient (*R^2^*) of ~0.97708 in 10 s response time. The existence of a good current response and reliable sensitivity might suggest high electron mobility and electrochemical activity [66] over the surface of HFCVD-grown WO_3_ NW thin-film electrodes. The stability performance of HFCVD-grown WO_3_ NW thin-film electrode materials was performed by a linear sweep voltammetry graph (current vs. voltage graph) in the presence of 20 μM methylamine. The electrochemical behavior was studied twice a day for 1 month. As seen in Figure 7b, ~95% of the current response remains the same as compared to the measurements performed on the first day of the analysis and no significant change in current is observed, which shows the good stability of HFCVD-grown WO_3_ NW thin-film electrodes. The performance of HFCVD-grown WO_3_ NW thin-film-based sensor is compared to other reported sensors [67,68,69,70,71], as shown in Table 1.

**Figure 8:**
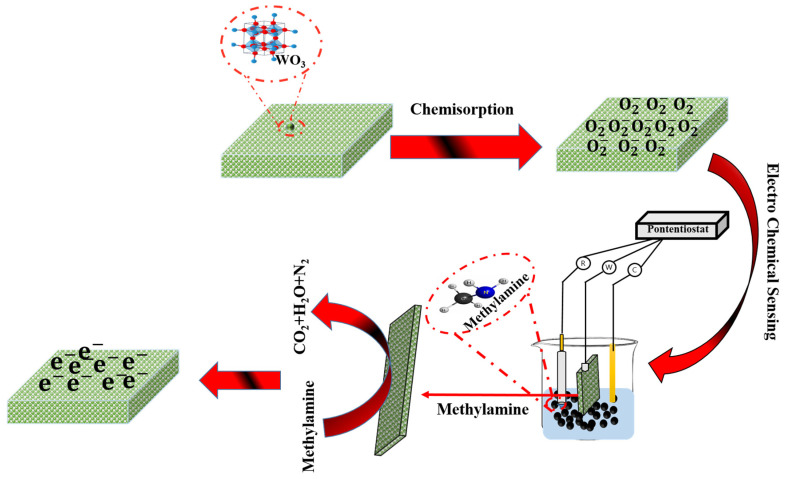
Possible sensing mechanism for the detection of methylamine over the surface of HFCVD-grown WO_3_ NW thin-film-based electrodes.

The detection mechanism of methylamine by HFCVD-grown WO_3_ NW thin-film-based electrodes is related to the changes in electrode conductance during the interaction of fabricated electrodes with an analyte. As shown in Figure 8, oxygen species adhere to the surface of WO_3_ NW thin films grown through the HFCVD process. The HFCVD-grown WO_3_ NW thin film shows n-type semiconducting behavior [33]. Due to the nature of WO_3_ thin films, electrons in the conduction band tend to bond with the surface area of WO_3_ thin films. By capturing electrons within WO_3_ thin films, these oxygen species (O_2_ adsorbed) transform into active sites, converting into anionic species containing oxygen [72]. The presence of these adsorbed oxygen species on the surface stimulates low-energy electrons in the valence band, leading to an increase in the number of holes within the HFCVD-grown WO_3_ NW thin film. This accumulation of holes eventually results in reduced resistance in the fabricated sensor. When the target methylamine molecule interacts with the adsorbed oxygen ions, it triggers the release of trapped electrons, as described by the following equations:

$$O_{2}\;(\mathrm{gas})↔O_{2}\;(\mathrm{ads})$$

$$O_{2}\;(\mathrm{ads})+ē\;(\mathrm{CB}\mathrm{of}{\mathrm{WO}}_{3})↔O_{2}^{-}\;(\mathrm{ads})\to O^{-}\;(\mathrm{ads})$$

$$8O^{-}+C_{2}H_{4}{\left(NH_{2}\right)}_{2}↔4CO_{2}+H_{2}O+2N_{2}+4ē$$

During the oxidation process, electrons emitted from the conduction band of the HFCVD-grown WO_3_ thin-film NWs enhance the electrical conductivity, resulting in an increased current associated with the methylamine [73]. In our work, the deposited WO_3_ thin film exhibits uniformly distributed nanowalls of an average grain size of ~20–25 nm. This structure might provide a large surface area for adsorption, making it highly efficient for the detection of methylamine, ensuring exceptional electrochemical performance.

## 4. Conclusions

This study focuses on the development of a sensitive sensor for detecting the hazardous chemical methylamine. A uniform WO_3_ thin-film nanowall structure on a Si substrate is obtained at a relatively low temperature of ~400 °C through HFCVD. The resulting WO_3_ NW-based thin-film-based electrode exhibits highly effective detection of methylamine at very low concentrations. The obtained results are attributed to the unique nanowall-like structure of WO_3_ thin films, which might offer a large surface area, facilitating efficient electron transfer during the electrochemical detection of methylamine. The WO_3_ NW-based fabricated chemical sensor demonstrates a promising sensitivity of ~196.33 μA μM^−1^ cm^−2^, a low limit of detection (LOD) of ~12 μM, and a strong retention coefficient of ~0.97708. Thus, our fabricated sensor has the potential for application in environmental monitoring, making it adaptable for the detection of other harmful chemicals in the future.

## Figures and Tables

**Table 1:** Sensing parameters of WO_3_ NWs electrode-based sensors compared with reported chemical sensors.

Materials	Preparation Method	Chemicals	Sensitivity	LOD	R^2^	Refs.
WO_3_	HFCVD	ethylenediamine	161.33 μA μM^−1^ cm^−2^	9.56 μM	0.98	[33]
PANI/Gr	Spin coating	hydrazine	32.54 × 10^−5^ μA cm^−2^ mM^−1^	15.38 mM	0.78578	[38]
Ag_2_O	Sonochemical method	acetone	1.689 μA cm^−2^ mM^−1^	0.11 μM	0.946	[67]
Ce_2_O_3_	Wet chemical method	2-nitrophenol	1.689 μA mM^−1^ cm^−2^		0.9030	[68]
WO_3_	HFCVD	diethylamine	3.5 μA μM^−1^ cm^−2^	7 μM		[69]
ZnFe_2_O_4_	Hydrothermal method	formaldehyde	4.10 μA cm^−2^ mM^−1^	0.89 μM		[70]
MAPbBr_3_	Electrospun	methylamine	-	0.8 ppm	0.9904	[71]
WO_3_	HFCVD	methylamine	183.65 μA μM^−1^ cm^−2^	20 μM	0.97708	This work

## Data Availability

The original contributions presented in the study are included in the article, further inquiries can be directed to the corresponding authors.
